# Isocaloric substitution with beef and mutton and metabolically unhealthy obesity among adults in Ordos, Inner Mongolia: a cross-sectional analysis

**DOI:** 10.3389/fnut.2026.1914471

**Published:** 2026-08-04

**Authors:** Yingjie Tian, Sha Du, Xinyan Wang, Yanling Wang, Huiqiu Zheng, Yanling Li, Hailin Li, Jingxia Fan, Jiahui Ding, Jiale Zhang, Yifan Liu, Xin Wang, Xuemei Wang

**Affiliations:** 1 Department of Epidemiology, School of Public Health, Shanxi Medical University, Taiyuan, China; 2 Department of Health Statistics, School of Public Health, Inner Mongolia Medical University, Hohhot, China; 3 Inner Mongolia People’s Hospital, Hohhot, China; 4 Health Big Data Research Center, Inner Mongolia Medical University, Hohhot, China; 5 Department of Child and Adolescent Health and Health Education, School of Public Health, Inner Mongolia Medical University, Hohhot, China; 6 School of Public Health, Baotou Medical College, Baotou, Inner Mongolia, China

**Keywords:** beef and mutton, food-group substitution, isocaloric substitution, metabolically healthy obesity, metabolically unhealthy obesity, nutritional epidemiology

## Abstract

**Background:**

Metabolic health differs substantially among adults with obesity, and dietary structure may contribute to this heterogeneity. In Ordos, Inner Mongolia, beef and mutton are common food sources within a livestock-oriented dietary setting rather than merely generic red-meat exposures.

**Methods:**

We analyzed 1,103 adults with obesity from the 2024 Ordos Region Residents’ Health White Paper survey. Dietary intake was assessed using an 83-item region-specific semi-quantitative food-frequency questionnaire and expressed as the percentage of total energy intake from 16 mutually exclusive food groups. Associations with metabolically unhealthy obesity (MUO) were evaluated using multivariable logistic regression, restricted cubic splines, and all-components isocaloric substitution models.

**Results:**

Overall, 440 (39.9%) were classified as metabolically healthy obesity (MHO) and 663 (60.1%) as MUO. In fully adjusted models, beef and mutton intake was inversely associated with MUO (OR per 5%E = 0.821, 95% CI: 0.719–0.933; *p* = 0.003). Compared with participants in the lowest quartile, those in the highest quartile of beef and mutton intake had lower odds of MUO (OR = 0.581, 95% CI: 0.394–0.856; *p*-for trend = 0.017). Restricted cubic spline analysis suggested an approximately linear inverse association (*p*-overall = 0.013; *p*-nonlinearity = 0.938). In theoretical substitution models, replacing selected food groups with beef and mutton was associated with lower odds of MUO. Dairy intake showed a weaker and less consistent inverse association.

**Conclusion:**

Among adults with obesity in Ordos, higher dietary energy from beef and mutton within the regional dietary structure was associated with lower odds of MUO in selected modeled substitution contrasts. These context-specific, cross-sectional findings require confirmation in prospective studies.

## 1 Introduction

Obesity is a major public health challenge and is closely associated with type 2 diabetes (T2D), cardiovascular disease, metabolic syndrome and several cancers (1). Its prevalence has increased over recent decades (2), and nearly 20% of the global population is projected to have obesity by 2030 (3). In China, overweight and obesity has also increased, with substantial regional heterogeneity and a higher prevalence in northern than in southern regions (4–6). Obesity, however, is not a metabolically uniform condition (7). Some adults with obesity maintain relatively normal glucose, lipid and blood-pressure profiles for a period and are classified as having MHO (8), whereas others have insulin resistance, dyslipidemia, elevated blood pressure, and related abnormalities and are classified as having MUO. Because MHO is not necessarily stable or benign (9), and may progress to MUO with higher cardiometabolic risk (10, 11), identifying modifiable determinants of metabolic phenotype among adults with obesity is important for precision prevention and nutritional management.

Inner Mongolia provides a distinctive setting in which to examine obesity-related metabolic heterogeneity. The prevalence of obesity in this region (38.5%) (12) is higher than the national average in China (11.24%) (13). In a previous survey in Ordos, however, 39.7% of adults with obesity were classified as MHO (14), a proportion higher than that reported in several other Chinese populations (11, 15, 16). The coexistence of a high obesity prevalence and a relatively high MHO proportion suggests that regional dietary and lifestyle factors may contribute to metabolic phenotypic differentiation beyond adiposity alone.

Diet is an important and modifiable determinant of metabolic health in obesity (17). Research on diet and chronic disease has increasingly moved beyond single nutrients or foods towards dietary patterns and diet-quality scores, which better capture habitual food combinations and their links with insulin resistance, low-grade inflammation and dyslipidemia (18). In Ordos, residents commonly consume diets characterized by high cereal intake and high livestock-meat intake, particularly beef and mutton. Meat consumption in this setting exceeds both global dietary-pattern estimates and Chinese dietary guideline recommendations (19). Previous work in this population identified a northern pastoral dietary pattern and showed that it was associated with obesity-related metabolic phenotypes (14). These findings suggest that local dietary features, including high beef and mutton intake, may be relevant to metabolic heterogeneity. Dietary-pattern analysis, however, does not answer a practical substitution question: under relatively stable total energy intake, which food group is lower when another food group is higher?

Isocaloric substitution modelling provides a framework for addressing this question. Within a fixed total-energy framework, higher intake of one food group generally implies lower intake of one or more other food groups (20, 21). This approach has been extended from nutrient-level analyses to food-group analyses that more closely reflect dietary decision making (22, 23). Leave-one-out and energy-partition models are commonly used for food-energy substitution and perform similarly when the target estimand is the replacement of one component by another measured on the same scale (24). When several components jointly contribute to total energy intake, however, simplified models may be biased. The all-components model includes the energy contribution of all major food groups simultaneously and estimates theoretical substitution contrasts by comparing regression coefficients between components (24, 25). Previous substitution studies have mainly examined replacement of red or processed meat with plant-based foods, legumes, whole grains or other protein sources, with outcomes such as T2D, cardiovascular disease and insulin sensitivity (26–28). Evidence remains limited for beef and mutton substitution in a northern pastoral Chinese dietary context, particularly in relation to obesity-related metabolic phenotypes.

We therefore examined the associations of major food-group energy contributions with MUO among adults with obesity in Ordos, Inner Mongolia, with particular attention to beef and mutton intake within a livestock-oriented dietary context. We further used an all-components isocaloric substitution framework to estimate theoretical replacement contrasts among major food groups under a fixed total-energy framework. The study was designed to provide context-specific nutritional epidemiology evidence and generate hypotheses for prospective and mechanistic research rather than to infer causal dietary effects.

## 2 Materials and methods

### 2.1 Study population

We analyzed data from a cross-sectional survey conducted as part of the 2024 Ordos Region Residents’ Health White Paper project. Adults aged 18 years or older were recruited using a multistage stratified random sampling approach. The minimum sample size was estimated using the formula:


$$n=\frac{z_{(1-\alpha )/2}^{2}p(1-p)}{d^{2}}$$

**Figure 1:**
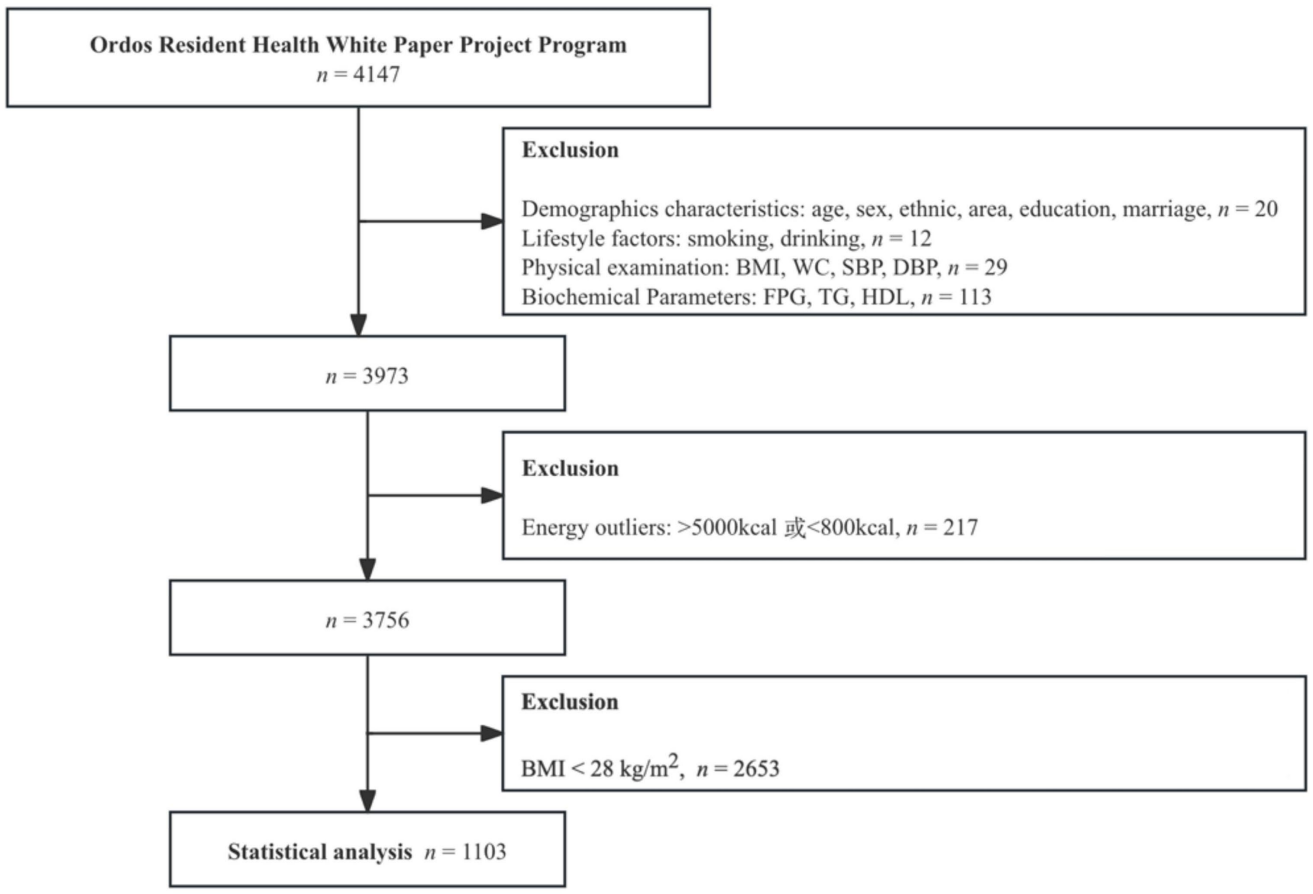
Flowchart of eligible participants in the study.

Based on a previously reported MUO prevalence of 60.3% (14) in this population, with *α* = 0.05 and an allowable error of *d* = 0.05, the minimum required sample size was estimated to be 368. The final analytic sample included 1,103 adults with obesity, which exceeded the minimum sample-size requirement (Figure 1).

Participants were excluded if they had missing key demographic, lifestyle, laboratory, anthropometric or dietary data, reported implausible energy intakes (<800 or >5,000 kcal/day), or had missing BMI data.

The study protocol was approved by the Biomedical Research Ethics Committee of Inner Mongolia Medical University (No. YKD202301132). All participants provided written informed consent before data collection.

### 2.2 Dietary exposure and food-group energy intake

Dietary intake was assessed in face-to-face interviews using an 83-item, region-specific semiquantitative food frequency questionnaire (FFQ). Participants recalled their dietary intake during the previous 30 days. For each item, they reported consumption status, frequency (times per day, week, month or year) and usual portion size. Standardized tableware, food models and pictorial aids were used to improve portion-size estimation and reduce recall error.

Average daily intake of each food item (g) was calculated as portion size (g) multiplied by frequency and divided by the number of days. Energy intake was estimated using the China Food Composition Tables (6th edition, 2019), supplemented by the 2004 and 2009 editions for items without values in the 2019 edition.

Food items were classified into 16 mutually exclusive food groups: refined grains, whole grains, legumes, tubers, vegetables and fruits, dairy, beef and mutton, other red meat, processed meat, poultry/fish/eggs, plant oil, animal oil, snacks, sugar-sweetened beverages (SSBs), alcohol and condiments. The energy contribution of each food group (%E) was calculated as energy from that group divided by total daily energy intake. For interpretability, food-group variables were modelled per 5% increment in total energy intake (5%E).

The 16 food groups were designed to capture nearly all FFQ-derived dietary energy. In the analytic sample, their summed energy contribution had a median of 100.0% and a mean of 99.65% (SD 1.31%). Because the food-group components were nearly compositional, substitution estimates were interpreted as theoretical replacement contrasts under a fixed total-energy framework. Model-rank diagnostics showed that the matrix including the 16 food groups and intercept was full rank in the analytic dataset. The high condition number nevertheless indicated that individual food-group coefficients should be interpreted through pre-specified substitution contrasts rather than as isolated independent effects.

### 2.3 Covariates

Covariates were selected *a priori* on the basis of previous literature and regional context. Age was calculated from participant-reported date of birth. Ethnicity was classified as Han, Mongolian or other. Residence was categorized as urban, rural or pastoral. Educational attainment was grouped as primary school or below, secondary education, and college or above. Marital status was classified as married, unmarried or other. Monthly household income was categorized as <3,000, 3,000–5,999, 6,000–8,999 or ≥9,000 RMB.

Smoking status was classified as never, former or current smoking. Alcohol consumption was categorized as non-drinking, moderate drinking or excessive drinking. Physical activity was assessed with the International Physical Activity Questionnaire Short Form (IPAQ-SF). Participants reported the frequency (days/week) and duration (minutes/day) of vigorous-intensity activity, moderate-intensity activity and walking during the previous 7 days.

Total physical activity was calculated as metabolic equivalent minutes per week (MET-min/week) according to the standardized IPAQ scoring protocol, assigning 8.0, 4.0 and 3.3 METs to vigorous activity, moderate activity and walking, respectively (29). In accordance with IPAQ recommendations, values exceeding 180 min/day for any activity domain were truncated. Total physical activity was then categorized as low (<600 MET-min/week), moderate (600–2,999 MET-min/week) or high (≥3,000 MET-min/week).

### 2.4 Anthropometric measurements and disease definition

Anthropometric measurements were performed by trained staff according to standardized protocols. Height and weight were measured to the nearest 0.1 cm and 0.1 kg, respectively, with participants wearing light clothing and no shoes. Waist and hip circumferences were measured twice, and mean values were used in the analyses.

Metabolic status was defined according to the updated National Cholesterol Education Program Adult Treatment Panel III criteria (30). MUO was defined as the presence of at least three of the following components among adults with obesity: (1) abdominal obesity, waist circumference ≥90 cm in men or ≥85 cm in women; (2) elevated triglycerides, ≥1.7 mmol/L; (3) reduced high-density lipoprotein cholesterol (HDL-C), <1.03 mmol/L in men or <1.29 mmol/L in women; (4) elevated blood pressure, systolic blood pressure (SBP) ≥130 mmHg and/or diastolic blood pressure (DBP) ≥85 mmHg, or current use of antihypertensive medication; and (5) elevated fasting plasma glucose (FPG), ≥6.1 mmol/L, glycated hemoglobin (HbA1c) ≥6.5%, or current use of glucose-lowering medication. MHO was defined as obesity (BMI ≥ 28.0 kg/m^2^) with 0–2 metabolic syndrome components.

### 2.5 Laboratory measurements

Fasting venous blood samples were collected after an overnight fast of at least 8 h. Samples were processed, stored and transported according to standardized protocols. Serum or plasma biochemical analyses included FPG, HbA1c, TG, TC, HDL-C and LDL-C. Quality assurance included daily internal quality checks and routine external quality assessment. Laboratory personnel were blinded to participants’ dietary data.

### 2.6 Isocaloric substitution analysis

We used the all-components model (24, 25) to estimate the associations for theoretical replacement between food groups and MUO. All 16 food groups were entered simultaneously as percentages of total energy intake (%E), together with covariates. Total energy intake was not included in the primary %E-based substitution model. Analyses with additional total-energy adjustment were treated as sensitivity analyses rather than as the primary specification. In this cross-sectional study, the model was used to estimate theoretical substitution contrasts and the estimates were interpreted as associations, not causal effects.

Specifically, all 16 food groups were entered simultaneously into a multivariable logistic regression model as percentages of total energy intake (%E):


$$\begin{array}{l} \text{logit}[P(\mathrm{MUO}=1)]=\beta_{0}+\beta_{1}({\text{Food}}_{1})\%E+\beta_{2}({\text{Food}}_{2})\%E+\\ \ldots +\beta_{16}({\text{Food}}_{16})\%E+\gamma^{\prime }X \end{array}$$

where ${\text{Food}}_{1}$ to ${\text{Food}}_{16}$ represent the 16 food groups, %*E* denotes the percentage of total energy intake contributed by each food group and *X* is a vector of covariates including age, sex, ethnicity, area, education, marital status, household income, smoking, drinking and physical activity.

The theoretical substitution contrast for replacing food group *B* with food group *A* was estimated as exp·[*kx* (*βA* − *βB*)], where *k* was 5 for the primary 5%E substitution analysis and 1 for the sensitivity 1%E substitution analysis.

### 2.7 Statistical analysis

Continuous variables were summarized as means and standard deviations (*SDs*) for approximately normally distributed data and as medians and interquartile ranges (IQRs) for skewed data. Categorical variables were summarized as *n* (%). Between-group comparisons used Student’s *t*-test, the Wilcoxon rank-sum test or the Chi-squared test, as appropriate.

Associations between dietary exposures and MUO were examined using binary logistic regression to estimate odds ratios (ORs) and 95% confidence intervals (CIs). Given the cross-sectional design, these estimates were interpreted as associations with the odds of MUO rather than as causal effects.

Restricted cubic spline (RCS) regression was used to examine dose–response associations, with three knots placed at the 10th, 50th and 90th percentiles of each exposure distribution (31). Likelihood ratio tests were used to evaluate overall and nonlinear associations.

Regression models were adjusted for age, sex, ethnicity, area, marital status, education, household income, smoking, drinking and physical activity. Statistical significance for overall spline associations was determined using the global *p*-value. Nonlinearity was considered present when the nonlinear *p*-value was <0.05.

Several sensitivity and diagnostic analyses were conducted to assess robustness and interpretability. First, leave-one-out analyses were used to determine whether the observed associations were driven by any single food group. Second, *E*-values were calculated to estimate the minimum strength of association that an unmeasured confounder would need to have with both the exposure and outcome to fully explain the observed associations. Third, substitution models were re-estimated using absolute food intake rather than percentage of energy intake as the exposure metric. Fourth, the primary substitution model was re-estimated with additional adjustment for total energy intake. Fifth, 1%E substitution analyses were performed because 5%E may be large for low-consumption food groups. Sixth, to reduce the possibility of reverse causation due to dietary changes after diagnosis or treatment of hypertension or diabetes, we conducted two additional sensitivity analyses. In the first analysis, we repeated the primary substitution models after excluding participants who reported current use of antihypertensive or glucose-lowering medication. In the second analysis, we redefined metabolic status using measured blood pressure and fasting plasma glucose values only, without considering medication use.

All statistical tests were two sided, and *p* < 0.05 was considered statistically significant. Data processing and basic statistical analyses were performed using SPSS version 26.0. Restricted cubic spline and substitution analyses were conducted using R version 4.5.1.

## 3 Results

### 3.1 Study population and characteristics of MHO and MUO

Figure 1 shows participant selection. The final analytic sample included 1,103 adults with obesity, of whom 440 (39.9%) were classified as MHO and 663 (60.1%) as MUO.

Table 1 presents baseline characteristics. Compared with participants with MHO, those with MUO were older and differed by ethnicity and residence. Smoking status and physical activity also differed between groups, whereas sex, education, marital status and income were broadly similar. As expected from the case definition, participants with MUO had higher BMI, waist circumference, HbA1c, fasting glucose, triglycerides, SBP and DBP, and lower HDL-C. Total cholesterol and LDL-C did not differ materially between groups.

**Table 1:** General demographic characteristics.

Characteristics		MHO (*n* = 440)	MUO (*n* = 663)	*p*-value
Age (years)		47.00 (38.00, 55.00)	49.00 (40.00, 57.50)	0.013
Sex	Male	272 (61.8)	440 (66.4)	0.138
	Female	168 (38.2)	223 (33.6)	
Ethnic	Han	336 (76.4)	551 (83.1)	0.015
	Mongolian	90 (20.5)	92 (13.9)	
	Others	14 (3.2)	20 (3.0)	
Area	Town	116 (26.4)	228 (34.4)	0.006
	Rural	266 (60.5)	375 (56.6)	
	Pastoral	58 (13.2)	60 (9.0)	
Education	<Primary	119 (27.0)	181 (27.3)	0.818
	Secondary	198 (45.0)	282 (42.5)	
	>College	123 (28.0)	200 (30.2)	
Marriage	Married	404 (91.8)	613 (92.5)	0.445
	Unmarried	22 (5.0)	24 (3.6)	
	Others	14 (3.2)	26 (3.9)	
Income month	<3,000	104 (23.7)	170 (25.6)	0.658
	3,000–6,000	117 (26.7)	169 (25.5)	
	6,000–9,000	100 (22.8)	163 (24.6)	
	>9,000	118 (26.9)	161 (24.3)	
Smoking	Never	252 (57.3)	343 (51.7)	0.040
	Current	146 (33.2)	269 (40.6)	
	Quit	42 (9.5)	51 (7.7)	
Drinking	Never	280 (63.6)	410 (61.8)	0.063
	Moderation	115 (26.1)	154 (23.2)	
	Excessive	45 (10.2)	99 (14.9)	
Physical activity	Low level	82 (18.6)	166 (25.0)	0.016
	Medium level	133 (30.2)	209 (31.5)	
	High level	225 (51.1)	288 (43.4)	
Antihypertensive drugs		97 (22.0)	278 (41.9)	<0.001
Antidiabetic drugs		4 (0.9)	72 (10.9)	<0.001
BMI (kg/m^2^)		29.97 (28.83, 31.57)	30.13 (29.01, 32.15)	0.024
WC		98.00 (93.00, 103.40)	100.00 (95.00, 104.20)	<0.001
HbA1c (%)		5.60 (5.40, 5.80)	6.20 (5.60, 7.50)	<0.001
FPG (mmol/L)		4.49 (4.11, 4.85)	4.67 (4.18, 5.49)	<0.001
TG (mmol/L)		1.34 (1.02, 1.64)	2.27 (1.75, 3.04)	<0.001
TC (mmol/L)		5.16 (4.53, 5.99)	5.21 (4.53, 6.00)	0.378
HDL-C (mmol/L)		1.26 (1.11, 1.43)	1.13 (0.97, 1.28)	<0.001
LDL-C (mmol/L)		3.20 (2.66, 3.75)	3.18 (2.68, 3.62)	0.486
SBP (mmHg)		125.00 (114.88, 139.12)	135.00 (126.50, 148.25)	<0.001
DBP (mmHg)		80.50 (72.50, 88.50)	88.00 (81.00, 95.00)	<0.001
Energy (kcal/day)		2174.38 (1718.35, 2835.30)	2224.00 (1727.09, 2841.55)	0.635

### 3.2 Dietary energy contributions

As shown in Table 2, total energy intake was comparable between the MHO and MUO groups, but the percentage of energy derived from several food groups differed. Compared with participants with MHO, those with MUO derived a greater proportion of energy from legumes, poultry/fish/eggs and condiments, and a smaller proportion from dairy products and beef/mutton. Most other food groups, including refined grains, other red meat, plant oil, animal oil, snacks, SSBs and alcohol, did not differ materially between groups. These findings indicate differences in dietary structure between MHO and MUO, although not across all major food sources.

**Table 2:** Percentage of energy intake from major food groups in MHO and MUO.

Dietary intake (E%)	MHO (*n* = 440)	MUO (*n* = 663)	*p-*value
Energy (kcal/day)	2174.38 (1718.35, 2835.30)	2224.00 (1727.09, 2841.55)	0.635
Refined grains	29.27 (22.19, 37.73)	29.39 (21.26, 39.96)	0.619
Whole grains	3.27 (0.87, 6.65)	3.72 (1.15, 7.47)	0.076
Legumes	1.89 (0.68, 3.91)	2.52 (0.92, 4.60)	0.005
Tubers	1.87 (0.41, 2.98)	1.97 (0.57, 3.26)	0.275
Vegetables and fruits	4.21 (2.64, 6.33)	4.43 (2.80, 6.36)	0.372
Dairy	3.95 (1.05, 10.02)	3.24 (0.43, 8.60)	0.025
Beef and mutton	3.70 (1.55, 8.03)	2.92 (1.08, 6.21)	<0.001
Other red meat	10.31 (4.47, 16.33)	10.20 (4.90, 16.55)	0.684
Processed meat	0.00 (0.00, 0.50)	0.00 (0.00, 0.62)	0.884
Poultry/fish/eggs	2.64 (1.15, 4.09)	2.91 (1.59, 4.43)	0.021
Plant oil	9.71 (6.17, 14.44)	9.62 (5.46, 14.27)	0.264
Animal oil	3.11 (0.00, 8.70)	3.78 (0.00, 10.10)	0.131
Snacks	2.24 (0.00, 5.56)	1.98 (0.00, 5.03)	0.276
SSBs	0.00 (0.00, 1.01)	0.00 (0.00, 1.17)	0.734
Alcohol	0.00 (0.00, 1.30)	0.00 (0.00, 1.69)	0.307
Condiments	0.20 (0.01, 0.88)	0.30 (0.01, 0.95)	0.025

### 3.3 Associations of individual food groups with MUO

In the fully adjusted model (Model 3) (Table 3), most food groups were not significantly associated with MUO. Beef and mutton intake showed an inverse association with MUO (OR per 5%E increment = 0.821, 95% CI: 0.719–0.933; *p* = 0.003). In quartile analysis (Table 4), participants in the highest quartile of beef and mutton intake had lower odds of MUO than those in the lowest quartile (OR = 0.581, 95% CI: 0.394–0.856; *p*-for trend = 0.017). Dairy intake was inversely associated with MUO in crude and partially adjusted models, but the association was attenuated and was no longer significant after full adjustment in continuous analysis (OR per 5%E increment = 0.960, 95% CI: 0.888–1.037; *p* = 0.297). No clear associations were observed for other food groups after multivariable adjustment.

**Table 3:** Associations of major food groups with MUO.

Dietary intake (5%E)	Model 1	Model 2	Model 3
Refined grains	1.014 (0.968, 1.061)	1.013 (0.967, 1.061)	1.023 (0.975, 1.074)
Whole grains	1.051 (0.951, 1.166)	1.042 (0.942, 1.156)	1.030 (0.927, 1.147)
Legumes	1.188 (1.015, 1.407) ^*^	1.210 (1.033, 1.436) ^*^	1.140 (0.968, 1.361)
Tubers	1.146 (0.844, 1.563)	1.136 (0.830, 1.563)	1.080 (0.779, 1.506)
Vegetables and fruits	1.032 (0.889, 1.204)	1.072 (0.921, 1.256)	1.064 (0.908, 1.253)
Dairy	0.937 (0.881, 0.995) ^*^	0.917 (0.861, 0.977) ^**^	0.960 (0.888, 1.037)
Beef and mutton	0.794 (0.709, 0.886) ^***^	0.778 (0.693, 0.870) ^***^	0.821 (0.719, 0.933) ^**^
Other red meat	0.996 (0.931, 1.066)	0.996 (0.931, 1.067)	0.954 (0.887, 1.027)
Processed meat	1.066 (0.695, 1.681)	1.186 (0.765, 1.908)	1.130 (0.718, 1.844)
Poultry/fish/eggs	1.213 (0.952, 1.557)	1.274 (0.994, 1.645)	1.160 (0.894, 1.515)
Plant oil	0.972 (0.901, 1.049)	0.977 (0.905, 1.055)	0.974 (0.900, 1.055)
Animal oil	1.095 (1.002, 1.199) ^*^	1.102 (1.006, 1.210) ^*^	1.087 (0.988, 1.199)
Snacks	0.942 (0.830, 1.072)	0.973 (0.854, 1.109)	0.947 (0.827, 1.086)
SSBs	1.014 (0.912, 1.131)	1.031 (0.924, 1.157)	1.032 (0.921, 1.162)
Alcohol	1.105 (0.987, 1.246)	1.102 (0.980, 1.250)	0.974 (0.806, 1.188)
Condiments	1.105 (0.648, 1.938)	1.124 (0.657, 1.980)	0.982 (0.562, 1.754)

Model 1 was unadjusted.

Model 2 was adjusted for age and sex.

Model 3 was further adjusted for ethnicity, area, education, marital status, household income, smoking, drinking and physical activity. In the continuous analyses, odds ratios represent the odds of MUO associated with each 5%E increment in the percentage of energy contribution from each food group.

*p < 0.05, **p < 0.01, ***p < 0.001.

**Table 4:** Odds ratios for MUO according to quartiles of beef and mutton and dairy intake.

Food group	Q1	Q2	Q3	Q4
Beef and mutton				
Model1	Ref	0.743 (0.525, 1.051)	0.827 (0.583, 1.172)	0.534 (0.378, 0.752) ^**^
Model2	Ref	0.724 (0.509, 1.029)	0.810 (0.569, 1.153)	0.497 (0.350, 0.703) ^***^
Model3	Ref	0.733 (0.511, 1.049)	0.839 (0.585, 1.202)	0.581 (0.394, 0.856) ^*^
Dairy				
Model1	Ref	0.757 (0.535, 1.069)	0.816 (0.577, 1.154)	0.639 (0.453, 0.900) ^*^
Model2	Ref	0.745 (0.526, 1.054)	0.775 (0.545, 1.099)	0.557 (0.389, 0.796) ^**^
Model3	Ref	0.728 (0.509, 1.038)	0.785 (0.546, 1.127)	0.675 (0.449, 1.014)

Model 1 was unadjusted.

Model 2 was adjusted for age and sex.

Model 3 was further adjusted for ethnicity, area, education, marital status, household income, smoking, drinking and physical activity.

Quartile cut-off values for the percentage of total energy intake were as follows: beef and mutton, Q1 ≤0.63%, Q2 ≤3.52%, Q3 ≤9.11%, and Q4 ≤73.13%; dairy, Q1 ≤1.22%, Q2 ≤3.18%, Q3 ≤6.80%, and Q4 ≤53.54%. Q1 was used as the reference group. *p < 0.05, **p < 0.01, ***p < 0.001.

**Figure 2:**
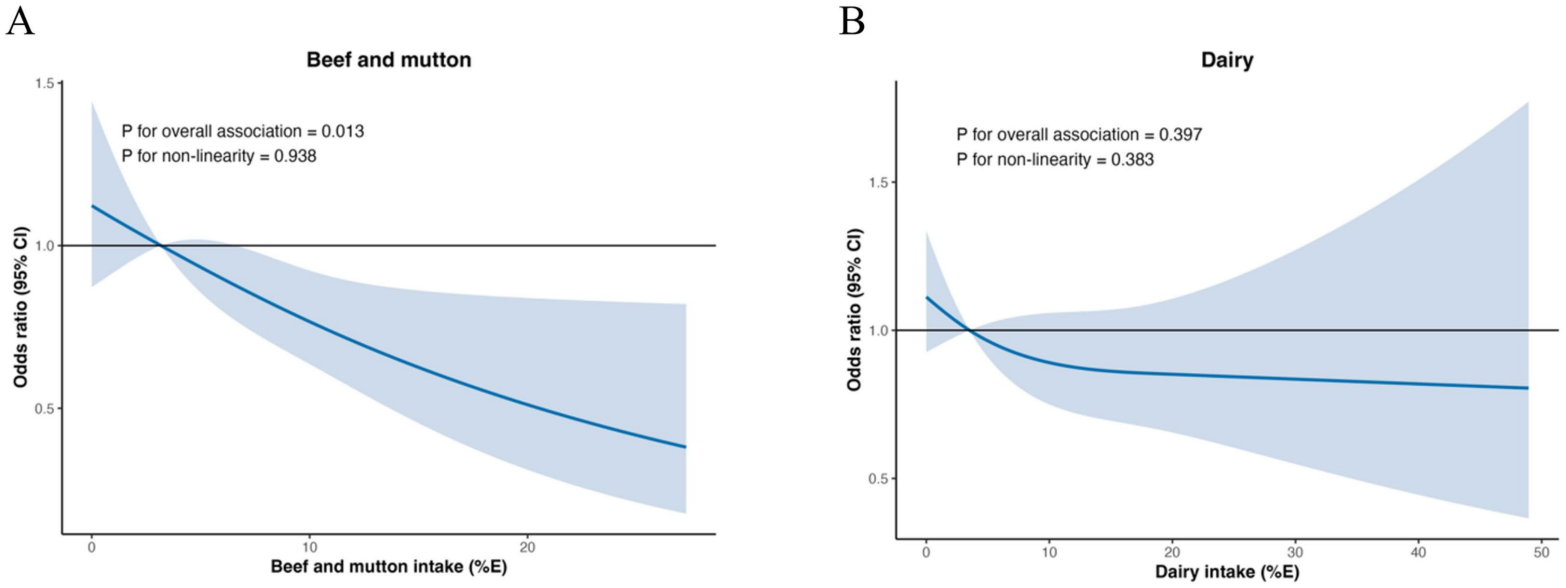
Dose-response associations of **(A)** beef and mutton intake and **(B)** dairy intake with MUO. Dose-response associations were assessed using restricted cubic spline logistic regression with three knots placed at the 10th, 50th, and 90th percentiles of the exposure distributions. The fully adjusted model included age, sex, ethnicity, area, education, marital status, household income, smoking, drinking, and physical activity. Solid lines indicate odds ratios, shaded areas indicate 95% confidence intervals, and the horizontal line indicates an odds ratio of 1.

Restricted cubic spline analysis showed an overall association between beef and mutton intake and MUO (*p-*overall = 0.013), with no evidence of nonlinearity (*p*-nonlinear = 0.938) (Figure 2). This pattern suggested an approximately linear inverse association across the observed intake range. No clear overall or nonlinear association was observed between dairy intake and MUO in the fully adjusted model (*p-*overall = 0.397; *p*-nonlinear = 0.383).

### 3.4 Isocaloric substitution analysis

**Figure 3:**
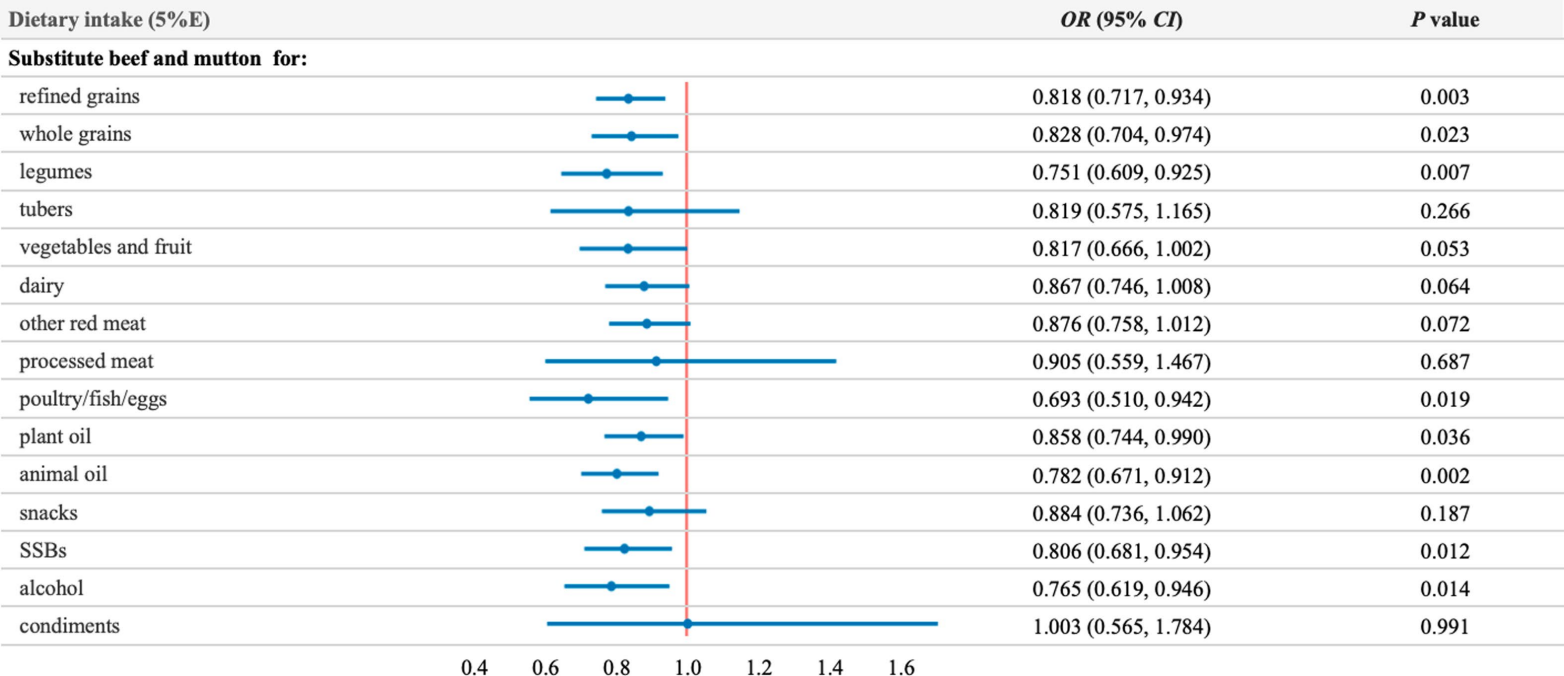
Associations of isocaloric substitution of beef and mutton with MUO. Associations were estimated using the all-components model and represent the odds of MUO associated with a 5%E increase in beef and mutton accompanied by an equivalent decrease in another food group. The primary model included all 16 food-group %E variables and adjusted for age, sex, ethnicity, residential area, education, marital status, household income, smoking, drinking and physical activity, without additional adjustment for total energy intake. Points indicate odds ratios, horizontal lines indicate 95% confidence intervals and the vertical line indicates an odds ratio of 1.

In fully adjusted isocaloric substitution models (Figure 3), theoretical replacement of several food groups with beef and mutton was associated with lower odds of MUO, particularly when replacing refined grains, whole grains, legumes, poultry/fish/eggs, animal oil, SSBs and alcohol. These substitution estimates are theoretical and reflect associations under model assumptions rather than causal effects of dietary replacement. The full substitution matrix is provided in the Supplementary materials.

### 3.5 Sensitivity analyses

Sensitivity analyses were performed to examine the robustness of the primary findings. First, leave-one-out analysis showed that the observed associations were not driven by any single food group (Supplementary Table 1). Second, *E*-values for the significant substitution associations ranged from 1.604 to 2.242, indicating that an unmeasured confounder would need to be associated with both the dietary substitution contrast and MUO by a risk ratio of at least this magnitude, conditional on measured covariates, to fully explain the observed associations (Supplementary Table 2). Third, when substitution models were re-estimated using absolute food intake rather than percentage of total energy intake, the direction of the associations was generally consistent with the primary analysis (Supplementary Table 3). Fourth, after additional adjustment for total energy intake, inverse associations remained statistically significant when beef and mutton theoretically replaced refined grains, whole grains, legumes, vegetables and fruits, poultry/fish/eggs, plant oil, animal oil, SSBs and alcohol (Supplementary Table 4). Fifth, analyses using 1%E substitution showed similar directions to the 5%E substitution models, although effect sizes were smaller as expected (Supplementary Table 5). Sixth, after excluding participants who reported current use of antihypertensive or glucose-lowering medication, inverse associations remained statistically significant when beef and mutton theoretically replaced refined grains, legumes, vegetables and fruits, poultry/fish/eggs, animal oil, SSBs, and alcohol (Supplementary Table 6). When MUO was redefined using measured blood pressure and fasting plasma glucose values only, inverse associations remained statistically significant for theoretical replacement of refined grains, legumes, vegetables and fruits, and SSBs with beef and mutton (Supplementary Table 7).

## 4 Discussion

The principal finding of this study was that, among adults with obesity in Ordos, a higher dietary energy contribution from beef and mutton was associated with lower odds of MUO. In theoretical substitution models, replacing refined grains, whole grains or legumes with beef and mutton was associated with lower odds of MUO. This association was observed in continuous, quartile, dose–response and modeled substitution analyses. Dairy intake tended to show an inverse direction, but the evidence was weaker after full adjustment and in sensitivity analyses. Overall, these findings suggest that the position of beef and mutton within the regional dietary structure of Ordos may be related to metabolic phenotypic differentiation among adults with obesity.

These findings should be interpreted in context of heterogeneous evidence on red meat and metabolic risk. Associations between processed meat, higher red meat intake and risk of T2D or MetS are relatively consistent. Gu et al. reported approximately linear positive associations of total red meat, processed red meat and unprocessed red meat with T2D risk in three large US cohorts (28). An umbrella review similarly suggested that high red meat intake, particularly processed meat intake, was associated with increased risk of several metabolic diseases (32). However, the evidence is not uniformly adverse. A systematic review of randomized controlled trials found no stable adverse effects of unprocessed lean red meat on fasting glucose, insulin or HbA1c (33). A Mendelian randomization study found no clear evidence for a causal relation between red meat intake and T2D (34). Burden of Proof analyses further rated the evidence linking unprocessed red meat with T2D and ischemic heart disease as weak, with limited average risk increases across typical exposure ranges (35). The present findings therefore do not simply contradict previous red-meat research. Rather, they underscore the need to distinguish processed from unprocessed meat, consider meat type and account for dietary context.

Differences in exposure and outcome definitions may also explain the apparent discrepancy with some prior red-meat studies. This study focused on beef and mutton rather than processed red meat or total red meat. In Ordos, beef and mutton are embedded in habitual dietary practices shaped by local livestock-oriented food preferences. Their preparation methods, co-consumed foods and eating contexts may differ from red-meat exposure in Western cohorts. The outcome was also MUO among adults with obesity, rather than incident T2D, cardiovascular disease or mortality in the general population. MUO reflects clustering of abnormal glucose, lipid and blood-pressure profiles in the context of obesity and may depend on overall energy allocation, carbohydrate quality, fat sources and lifestyle background. A multinational cohort analysis showed that replacing processed meat with unprocessed red meat or poultry was associated with lower T2D incidence (36). This supports the view that the health relevance of meat depends not only on the broad label of red meat but also on the specific meat source and the foods it replaces.

The isocaloric substitution framework further supports this interpretation. In nutritional epidemiology, substitution analysis recognizes that, under relatively fixed total energy intake, higher intake of one food group usually implies lower intake of another. The health relevance of a food therefore depends on what it replaces. Previous red-meat substitution studies have shown that replacing red or processed meat with plant-based foods, legumes, dairy products or other protein sources does not yield identical associations with T2D risk (26, 28, 37). Randomized trials and network meta-analyses have also suggested that the effects of red meat on lipid, glycemic and inflammatory markers vary according to the comparison diet (38, 39). The inverse association between beef and mutton and MUO in this study should therefore be understood as a relative association within specific substitution contrasts.

The substitution of refined grains and animal oil with beef and mutton is relatively interpretable. Higher refined-grain intake generally implies a greater load of rapidly available carbohydrate. In Indian adults, high refined-grain intake was strongly associated with higher MetS risk. Animal oil may increase saturated-fat and total-fat exposure. Saturated fatty acid-related ceramide accumulation has also been linked to impaired insulin signaling and lipotoxicity (40). In adults with obesity, these factors may jointly promote insulin resistance, dyslipidemia and elevated blood pressure.

The findings for whole grains, legumes and poultry/fish/eggs require more cautious interpretation. Whole grains are generally considered beneficial for insulin sensitivity and glucose metabolism, and a randomized trial in adults with obesity showed that a whole-grain diet improved peripheral insulin resistance and glucose kinetics (17). The inverse association observed when beef and mutton replaced whole grains should therefore not be interpreted as evidence that beef and mutton are superior to whole grains. It may instead reflect low local whole-grain intake, food classification differences, cooking methods, co-consumed foods or residual confounding. The legume result should be interpreted similarly. Some studies suggest the possibility that replacing red meat with legumes or plant-based foods may reduce T2D risk (26), whereas the EPIC-InterAct study did not observe a clear inverse association when legumes replaced red or processed meat (37). The present finding that replacing legumes with beef and mutton was associated with lower MUO is therefore more likely to reflect local forms of legumes and soybean products, processing methods, oil and salt use, staple-food combinations and participant selection factors than an adverse metabolic effect of legumes per se.

Biological plausibility can be considered, but should not be overstated. Beef and mutton are ruminant meats and contain ruminant-derived fatty acids, including trans-vaccenic acid and conjugated linoleic acid (c9t11-CLA). Experimental studies suggest that vaccenic acid may affect PPAR-*α*, PPAR-*γ* and insulin-secretion-related pathways (41, 42), and that combined CLA and vaccenic acid interventions may improve lipid metabolism in animal models (43). These data mainly come from animal or mechanistic studies and should be treated as hypothesis-generating background rather than evidence that beef and mutton directly reduce MUO through these pathways.

This study has several limitations. First, the cross-sectional design precludes assessment of the temporal relation between dietary intake and MUO and cannot exclude reverse causation from diet changes after metabolic abnormalities developed. Second, dietary intake was assessed using a self-reported FFQ covering the previous 30 days. Although trained interviewers used standardized tableware, food models and pictorial aids to improve portion-size estimation, recall bias and measurement error remain possible because participants were not asked to provide weighed food records or prospective dietary records. Third, substitution models estimate theoretical food-group replacement contrasts rather than actual intervention effects. Although sensitivity analyses showed generally consistent directions, causal inference remains limited. Fourth, the study was conducted in Ordos, Inner Mongolia, where dietary structure, ethnic composition and the food environment are regionally distinctive. The findings should therefore be interpreted primarily within this local dietary context and require external validation.

In conclusion, among adults with obesity in Ordos, higher dietary energy from beef and mutton was associated with lower odds of MUO, and this association was observed in several theoretical food-group substitution analyses. These findings should be interpreted as context-specific, cross-sectional associations within a regional livestock-oriented dietary pattern, rather than as evidence that beef and mutton reduce cardio-metabolic disease risk. Prospective studies with repeated dietary assessment and detailed information on meat processing, cooking methods and co-consumed foods are needed to clarify the temporal and causal relevance of these associations.

## Data Availability

The data, code book, and analytical code used in this manuscript are available from the corresponding author upon reasonable request.
