# Supplementary material for: Isocaloric substitution with beef and mutton and metabolically unhealthy obesity among adults in Ordos, Inner Mongolia: a cross-sectional analysis
Source: Front Nutr. 2026 Aug 4;13:1914471. doi: 10.3389/fnut.2026.1914471 (PMC13481200; doi:10.3389/fnut.2026.1914471)
Supplement: Supplementary file 2 [file Table_2.DOCX]

***Supplementary Material***

**Isocaloric substitution with beef and mutton and metabolically unhealthy obesity among adults in Ordos, Inner Mongolia: a cross-sectional analysis**

**Supplementary Tables**

**Supplementary Table 1. Leave-one-out analysis for modeled isocaloric substitution with beef and mutton.**

| **Substitution** | **Beta** | **SE** | **OR** | **Lower CI** | **Upper CI** | **OR (95% CI)** | **P value** |
| --- | --- | --- | --- | --- | --- | --- | --- |
| Beef and mutton replacing Refined grains | -0.042 | 0.013 | 0.809 | 0.709 | 0.924 | 0.809 (0.709, 0.924) | 0.002 |
| Beef and mutton replacing Whole grains | -0.040 | 0.016 | 0.817 | 0.695 | 0.96 | 0.817 (0.695, 0.960) | 0.014 |
| Beef and mutton replacing Legumes | -0.059 | 0.021 | 0.743 | 0.608 | 0.909 | 0.743 (0.608, 0.909) | 0.004 |
| Beef and mutton replacing Tubers | -0.056 | 0.031 | 0.754 | 0.558 | 1.019 | 0.754 (0.558, 1.019) | 0.066 |
| Beef and mutton replacing Vegetables and fruits | -0.048 | 0.020 | 0.786 | 0.647 | 0.956 | 0.786 (0.647, 0.956) | 0.016 |
| Beef and mutton replacing Dairy | -0.031 | 0.015 | 0.857 | 0.739 | 0.994 | 0.857 (0.739, 0.994) | 0.041 |
| Beef and mutton replacing Other red meat | -0.029 | 0.015 | 0.866 | 0.75 | 1. 000 | 0.866 (0.750, 1.000) | 0.051 |
| Beef and mutton replacing Processed meat | -0.051 | 0.035 | 0.774 | 0.549 | 1.091 | 0.774 (0.549, 1.091) | 0.144 |
| Beef and mutton replacing Poultry/fish/eggs | -0.081 | 0.028 | 0.666 | 0.505 | 0.879 | 0.666 (0.505, 0.879) | 0.004 |
| Beef and mutton replacing Plant oil | -0.034 | 0.015 | 0.842 | 0.729 | 0.973 | 0.842 (0.729, 0.973) | 0.02 |
| Beef and mutton replacing Animal oil | -0.052 | 0.016 | 0.769 | 0.66 | 0.897 | 0.769 (0.660, 0.897) | 0.001 |
| Beef and mutton replacing Snacks | -0.029 | 0.018 | 0.867 | 0.726 | 1.036 | 0.867 (0.726, 1.036) | 0.116 |
| Beef and mutton replacing Sugar-sweetened beverages | -0.045 | 0.017 | 0.799 | 0.676 | 0.943 | 0.799 (0.676, 0.943) | 0.008 |
| Beef and mutton replacing Alcohol | -0.059 | 0.021 | 0.744 | 0.606 | 0.913 | 0.744 (0.606, 0.913) | 0.005 |
| Beef and mutton replacing Condiments | -0.051 | 0.039 | 0.774 | 0.527 | 1.137 | 0.774 (0.527, 1.137) | 0.191 |

**Supplementary Table 2. E-values for significant modeled substitution associations.**

| **Substitution** | **OR (95% CI)** | **P value** | **E-value** |
| --- | --- | --- | --- |
| Increase Beef and mutton and decrease Refined grains | 0.818 (0.717, 0.934) | 0.003 | 1.74 |
| Increase Beef and mutton and decrease Whole grains | 0.828 (0.704, 0.974) | 0.023 | 1.708 |
| Increase Beef and mutton and decrease Legumes | 0.751 (0.609, 0.925) | 0.007 | 1.996 |
| Increase Beef and mutton and decrease Tubers | 0.819 (0.575, 1.165) | 0.266 | 1.74 |
| Increase Beef and mutton and decrease Vegetables and fruits | 0.817 (0.666, 1.002) | 0.053 | 1.747 |
| Increase Beef and mutton and decrease Dairy | 0.867 (0.746, 1.008) | 0.064 | 1.574 |
| Increase Beef and mutton and decrease Other red meat | 0.876 (0.758, 1.012) | 0.072 | 1.543 |
| Increase Beef and mutton and decrease Processed meat | 0.905 (0.559, 1.467) | 0.687 | 1.445 |
| Increase Beef and mutton and decrease Poultry/fish/eggs | 0.693 (0.510, 0.942) | 0.019 | 2.242 |
| Increase Beef and mutton and decrease Plant oil | 0.858 (0.744, 0.990) | 0.036 | 1.604 |
| Increase Beef and mutton and decrease Animal oil | 0.782 (0.671, 0.912) | 0.002 | 1.875 |
| Increase Beef and mutton and decrease Snacks | 0.884 (0.736, 1.062) | 0.187 | 1.516 |
| Increase Beef and mutton and decrease Sugar-sweetened beverages | 0.806 (0.681, 0.954) | 0.012 | 1.787 |
| Increase Beef and mutton and decrease Alcohol | 0.765 (0.619, 0.946) | 0.014 | 1.94 |
| Increase Beef and mutton and decrease Condiments | 1.003 (0.565, 1.784) | 0.991 | 1.057 |

**Supplementary Table 3. Modeled food-group substitution using absolute food intake.**

| **Substitution** | **Beta substitution** | **SE substitution** | **OR** | **Lower CI** | **Upper CI** | **OR (95% CI)** | **P value** |
| --- | --- | --- | --- | --- | --- | --- | --- |
| Increase Beef and mutton and decrease Refined grains | -0.002 | 0.001 | 0.992 | 0.987 | 0.998 | 0.992 (0.987, 0.998) | 0.006 |
| Increase Beef and mutton and decrease Whole grains | -0.002 | 0.001 | 0.992 | 0.986 | 0.999 | 0.992 (0.986, 0.999) | 0.026 |
| Increase Beef and mutton and decrease Legumes | -0.002 | 0.001 | 0.992 | 0.984 | 1. 000 | 0.992 (0.984, 1.000) | 0.060 |
| Increase Beef and mutton and decrease Tubers | -0.002 | 0.002 | 0.99 | 0.975 | 1.005 | 0.990 (0.975, 1.005) | 0.174 |
| Increase Beef and mutton and decrease Vegetables and fruits | -0.002 | 0.001 | 0.991 | 0.983 | 0.999 | 0.991 (0.983, 0.999) | 0.035 |
| Increase Beef and mutton and decrease Dairy | -0.001 | 0.001 | 0.995 | 0.989 | 1.001 | 0.995 (0.989, 1.001) | 0.088 |
| Increase Beef and mutton and decrease Other red meat | -0.001 | 0.001 | 0.995 | 0.989 | 1.001 | 0.995 (0.989, 1.001) | 0.081 |
| Increase Beef and mutton and decrease Processed meat | -0.001 | 0.002 | 0.994 | 0.974 | 1.015 | 0.994 (0.974, 1.015) | 0.591 |
| Increase Beef and mutton and decrease Poultry/fish/eggs | -0.004 | 0.001 | 0.982 | 0.969 | 0.995 | 0.982 (0.969, 0.995) | 0.008 |
| Increase Beef and mutton and decrease Plant oil | -0.001 | 0.001 | 0.995 | 0.989 | 1. 000 | 0.995 (0.989, 1.000) | 0.066 |
| Increase Beef and mutton and decrease Animal oil | -0.002 | 0.001 | 0.991 | 0.985 | 0.998 | 0.991 (0.985, 0.998) | 0.009 |
| Increase Beef and mutton and decrease Snacks | -0.001 | 0.001 | 0.995 | 0.988 | 1.003 | 0.995 (0.988, 1.003) | 0.209 |
| Increase Beef and mutton and decrease Sugar-sweetened beverages | -0.002 | 0.001 | 0.992 | 0.986 | 0.999 | 0.992 (0.986, 0.999) | 0.019 |
| Increase Beef and mutton and decrease Alcohol | -0.002 | 0.001 | 0.988 | 0.980 | 0.996 | 0.988 (0.980, 0.996) | 0.004 |
| Increase Beef and mutton and decrease Condiments | -0. 000 | 0.003 | 0.998 | 0.972 | 1.024 | 0.998 (0.972, 1.024) | 0.858 |

**Supplementary Table 4. Modeled isocaloric substitution additionally adjusted for total energy intake.**

| **Substitution** | **Beta substitution** | **SE substitution** | **OR** | **Lower CI** | **Upper CI** | **OR (95% CI)** | **P value** |
| --- | --- | --- | --- | --- | --- | --- | --- |
| Increase Beef and mutton and decrease Refined grains | -0.041 | 0.014 | 0.814 | 0.713 | 0.929 | 0.814 (0.713, 0.929) | 0.002 |
| Increase Beef and mutton and decrease Whole grains | -0.038 | 0.017 | 0.827 | 0.703 | 0.974 | 0.827 (0.703, 0.974) | 0.023 |
| Increase Beef and mutton and decrease Legumes | -0.056 | 0.021 | 0.756 | 0.613 | 0.933 | 0.756 (0.613, 0.933) | 0.009 |
| Increase Beef and mutton and decrease Tubers | -0.043 | 0.036 | 0.805 | 0.565 | 1.148 | 0.805 (0.565, 1.148) | 0.231 |
| Increase Beef and mutton and decrease Vegetables and fruits | -0.043 | 0.021 | 0.807 | 0.657 | 0.992 | 0.807 (0.657, 0.992) | 0.041 |
| Increase Beef and mutton and decrease Dairy | -0.028 | 0.015 | 0.871 | 0.749 | 1.013 | 0.871 (0.749, 1.013) | 0.073 |
| Increase Beef and mutton and decrease Other red meat | -0.027 | 0.015 | 0.875 | 0.756 | 1.011 | 0.875 (0.756, 1.011) | 0.070 |
| Increase Beef and mutton and decrease Processed meat | -0.02 | 0.049 | 0.904 | 0.559 | 1.464 | 0.904 (0.559, 1.464) | 0.683 |
| Increase Beef and mutton and decrease Poultry/fish/eggs | -0.08 | 0.032 | 0.670 | 0.491 | 0.916 | 0.670 (0.491, 0.916) | 0.012 |
| Increase Beef and mutton and decrease Plant oil | -0.033 | 0.015 | 0.848 | 0.734 | 0.980 | 0.848 (0.734, 0.980) | 0.026 |
| Increase Beef and mutton and decrease Animal oil | -0.051 | 0.016 | 0.775 | 0.664 | 0.905 | 0.775 (0.664, 0.905) | 0.001 |
| Increase Beef and mutton and decrease Snacks | -0.023 | 0.019 | 0.893 | 0.743 | 1.074 | 0.893 (0.743, 1.074) | 0.231 |
| Increase Beef and mutton and decrease Sugar-sweetened beverages | -0.042 | 0.017 | 0.811 | 0.684 | 0.961 | 0.811 (0.684, 0.961) | 0.015 |
| Increase Beef and mutton and decrease Alcohol | -0.056 | 0.022 | 0.757 | 0.612 | 0.938 | 0.757 (0.612, 0.938) | 0.011 |
| Increase Beef and mutton and decrease Condiments | -0.008 | 0.059 | 0.962 | 0.539 | 1.717 | 0.962 (0.539, 1.717) | 0.897 |

**Supplementary Table 5. Modeled 1%E isocaloric substitution with beef and mutton.**

| **Substitution** | **Beta substitution** | **SE substitution** | **OR** | **Lower CI** | **Upper CI** | **OR (95% CI)** | **P value** |
| --- | --- | --- | --- | --- | --- | --- | --- |
| Increase Beef and mutton and decrease Refined grains | -0.04 | 0.013 | 0.961 | 0.936 | 0.986 | 0.961 (0.936, 0.986) | 0.003 |
| Increase Beef and mutton and decrease Whole grains | -0.038 | 0.017 | 0.963 | 0.932 | 0.995 | 0.963 (0.932, 0.995) | 0.023 |
| Increase Beef and mutton and decrease Legumes | -0.057 | 0.021 | 0.944 | 0.905 | 0.985 | 0.944 (0.905, 0.985) | 0.007 |
| Increase Beef and mutton and decrease Tubers | -0.04 | 0.036 | 0.961 | 0.895 | 1.031 | 0.961 (0.895, 1.031) | 0.266 |
| Increase Beef and mutton and decrease Vegetables and fruits | -0.04 | 0.021 | 0.960 | 0.922 | 1.000 | 0.960 (0.922, 1.000) | 0.053 |
| Increase Beef and mutton and decrease Dairy | -0.028 | 0.015 | 0.972 | 0.943 | 1.002 | 0.972 (0.943, 1.002) | 0.064 |
| Increase Beef and mutton and decrease Other red meat | -0.027 | 0.015 | 0.974 | 0.946 | 1.002 | 0.974 (0.946, 1.002) | 0.072 |
| Increase Beef and mutton and decrease Processed meat | -0.02 | 0.049 | 0.98 | 0.890 | 1.08 | 0.980 (0.890, 1.080) | 0.687 |
| Increase Beef and mutton and decrease Poultry/fish/eggs | -0.073 | 0.031 | 0.929 | 0.874 | 0.988 | 0.929 (0.874, 0.988) | 0.019 |
| Increase Beef and mutton and decrease Plant oil | -0.031 | 0.015 | 0.970 | 0.942 | 0.998 | 0.970 (0.942, 0.998) | 0.036 |
| Increase Beef and mutton and decrease Animal oil | -0.049 | 0.016 | 0.952 | 0.923 | 0.982 | 0.952 (0.923, 0.982) | 0.002 |
| Increase Beef and mutton and decrease Snacks | -0.025 | 0.019 | 0.976 | 0.94 | 1.012 | 0.976 (0.940, 1.012) | 0.187 |
| Increase Beef and mutton and decrease Sugar-sweetened beverages | -0.043 | 0.017 | 0.958 | 0.926 | 0.991 | 0.958 (0.926, 0.991) | 0.012 |
| Increase Beef and mutton and decrease Alcohol | -0.054 | 0.022 | 0.948 | 0.908 | 0.989 | 0.948 (0.908, 0.989) | 0.014 |
| Increase Beef and mutton and decrease Condiments | 0.001 | 0.059 | 1.001 | 0.892 | 1.123 | 1.001 (0.892, 1.123) | 0.991 |

**Supplementary Table 6. Modeled 5%E isocaloric substitution with beef and mutton excluding participants using antihypertensive or glucose-lowering medication.**

| **Substitution** | **Beta substitution** | **SE substitution** | **OR** | **Lower CI** | **Upper CI** | **OR (95% CI)** | **P value** |
| --- | --- | --- | --- | --- | --- | --- | --- |
| Increase Beef and mutton and decrease Refined grains | -0.039 | 0.016 | 0.822 | 0.702 | 0.962 | 0.822 (0.702, 0.962) | 0.015 |
| Increase Beef and mutton and decrease Whole grains | -0.024 | 0.021 | 0.888 | 0.721 | 1.094 | 0.888 (0.721, 1.094) | 0.264 |
| Increase Beef and mutton and decrease Legumes | -0.067 | 0.026 | 0.715 | 0.555 | 0.922 | 0.715 (0.555, 0.922) | 0.010 |
| Increase Beef and mutton and decrease Tubers | -0.064 | 0.045 | 0.728 | 0.468 | 1.132 | 0.728 (0.468, 1.132) | 0.159 |
| Increase Beef and mutton and decrease Vegetables and fruits | -0.053 | 0.025 | 0.767 | 0.601 | 0.978 | 0.767 (0.601, 0.978) | 0.032 |
| Increase Beef and mutton and decrease Dairy | -0.024 | 0.019 | 0.886 | 0.735 | 1.068 | 0.886 (0.735, 1.068) | 0.204 |
| Increase Beef and mutton and decrease Other red meat | -0.019 | 0.018 | 0.911 | 0.763 | 1.088 | 0.911 (0.763, 1.088) | 0.305 |
| Increase Beef and mutton and decrease Processed meat | -0.014 | 0.059 | 0.930 | 0.521 | 1.661 | 0.930 (0.521, 1.661) | 0.807 |
| Increase Beef and mutton and decrease Poultry/fish/eggs | -0.086 | 0.037 | 0.652 | 0.453 | 0.939 | 0.652 (0.453, 0.939) | 0.021 |
| Increase Beef and mutton and decrease Plant oil | -0.017 | 0.018 | 0.920 | 0.771 | 1.099 | 0.920 (0.771, 1.099) | 0.359 |
| Increase Beef and mutton and decrease Animal oil | -0.045 | 0.020 | 0.798 | 0.657 | 0.969 | 0.798 (0.657, 0.969) | 0.023 |
| Increase Beef and mutton and decrease Snacks | -0.026 | 0.023 | 0.876 | 0.697 | 1.102 | 0.876 (0.697, 1.102) | 0.260 |
| Increase Beef and mutton and decrease Sugar-sweetened beverages | -0.045 | 0.020 | 0.800 | 0.656 | 0.976 | 0.800 (0.656, 0.976) | 0.028 |
| Increase Beef and mutton and decrease Alcohol | -0.050 | 0.024 | 0.777 | 0.614 | 0.984 | 0.777 (0.614, 0.984) | 0.036 |
| Increase Beef and mutton and decrease Condiments | 0.021 | 0.070 | 1.111 | 0.562 | 2.195 | 1.111 (0.562, 2.195) | 0.762 |

**Supplementary Table 7. Modeled 5%E isocaloric substitution with beef and mutton using MUO defined by measured blood pressure and fasting plasma glucose values only.**

| **Substitution** | **Beta substitution** | **SE substitution** | **OR** | **Lower CI** | **Upper CI** | **OR (95% CI)** | **P value** |
| --- | --- | --- | --- | --- | --- | --- | --- |
| Increase Beef and mutton and decrease Refined grains | -0.033 | 0.014 | 0.850 | 0.743 | 0.972 | 0.850 (0.743, 0.972) | 0.018 |
| Increase Beef and mutton and decrease Whole grains | -0.031 | 0.017 | 0.857 | 0.728 | 1.008 | 0.857 (0.728, 1.008) | 0.062 |
| Increase Beef and mutton and decrease Legumes | -0.043 | 0.021 | 0.808 | 0.660 | 0.988 | 0.808 (0.660, 0.988) | 0.038 |
| Increase Beef and mutton and decrease Tubers | -0.031 | 0.035 | 0.858 | 0.608 | 1.211 | 0.858 (0.608, 1.211) | 0.383 |
| Increase Beef and mutton and decrease Vegetables and fruits | -0.045 | 0.021 | 0.800 | 0.653 | 0.981 | 0.800 (0.653, 0.981) | 0.032 |
| Increase Beef and mutton and decrease Dairy | -0.016 | 0.016 | 0.923 | 0.790 | 1.077 | 0.923 (0.790, 1.077) | 0.309 |
| Increase Beef and mutton and decrease Other red meat | -0.018 | 0.015 | 0.913 | 0.789 | 1.058 | 0.913 (0.789, 1.058) | 0.227 |
| Increase Beef and mutton and decrease Processed meat | -0.009 | 0.048 | 0.956 | 0.596 | 1.533 | 0.956 (0.596, 1.533) | 0.851 |
| Increase Beef and mutton and decrease Poultry/fish/eggs | -0.030 | 0.031 | 0.862 | 0.638 | 1.164 | 0.862 (0.638, 1.164) | 0.332 |
| Increase Beef and mutton and decrease Plant oil | -0.027 | 0.015 | 0.873 | 0.755 | 1.010 | 0.873 (0.755, 1.010) | 0.069 |
| Increase Beef and mutton and decrease Animal oil | -0.028 | 0.016 | 0.869 | 0.746 | 1.014 | 0.869 (0.746, 1.014) | 0.074 |
| Increase Beef and mutton and decrease Snacks | -0.011 | 0.019 | 0.946 | 0.786 | 1.138 | 0.946 (0.786, 1.138) | 0.555 |
| Increase Beef and mutton and decrease Sugar-sweetened beverages | -0.038 | 0.017 | 0.828 | 0.699 | 0.981 | 0.828 (0.699, 0.981) | 0.029 |
| Increase Beef and mutton and decrease Alcohol | -0.038 | 0.021 | 0.827 | 0.674 | 1.013 | 0.827 (0.674, 1.013) | 0.067 |
| Increase Beef and mutton and decrease Condiments | 0.041 | 0.060 | 1.226 | 0.681 | 2.207 | 1.226 (0.681, 2.207) | 0.498 |
