# Supplementary material for: Isocaloric substitution with beef and mutton and metabolically unhealthy obesity among adults in Ordos, Inner Mongolia: a cross-sectional analysis
Source: Front Nutr. 2026 Aug 4;13:1914471. doi: 10.3389/fnut.2026.1914471 (PMC13481200; doi:10.3389/fnut.2026.1914471)
Supplement: Supplementary file 3 [file Table_3.DOCX]

**Response to Editor and Reviewer**

**Dear Editor and Reviewer,**

We sincerely appreciate your time and effort in reviewing our manuscript and providing us with the opportunity to improve the quality and clarity of our work. We have carefully considered all comments raised by the reviewers and have revised the manuscript accordingly.

We have provided a point-by-point response below and revised the corresponding parts of the manuscript and Supplementary Material. All revised text has been highlighted in red for ease of review. We hope that the revisions and responses satisfactorily address the reviewers’ concerns.

Thank you again for your time and consideration. We look forward to hearing from you.

Sincerely yours,

Xuemei Wang.

Manuscript ID: 1914471

Title: Isocaloric substitution with beef and mutton and metabolically unhealthy obesity among adults in Ordos, Inner Mongolia: a cross-sectional analysis

**Response to Reviewer 1**

## Point 1

**Reviewer comment:** The definition of MUO counts current use of blood-pressure and glucose-lowering medication. Participants who were already diagnosed and treated may have changed their diet as a result of their diagnosis. The reviewer asks the authors to (a) first report how many participants in each group were taking antihypertensive or glucose-lowering medication. (b) If the numbers allow, reviewer would then suggest a sensitivity analysis that reduces the risk of reverse causation. For example, repeating the main analysis after excluding those on such medication, or re-defining MUO using measured blood pressure and glucose values only. If the association still holds, this would strengthen confidence in the finding

**Response:** We thank the reviewer for raising this important point. We agree that participants receiving antihypertensive or glucose-lowering treatment may have changed their diet after diagnosis, which could introduce reverse causation in this cross-sectional analysis. To address this concern, we first added the numbers and percentages of participants using antihypertensive medication and antidiabetic drugs in **Table 1**. **(Line 555, Page 14)**

Second, we performed two additional sensitivity analyses. In the first analysis, we repeated the substitution models after excluding participants who reported current use of antihypertensive or glucose-lowering medication. In the second analysis, we redefined MUO using measured blood pressure and fasting plasma glucose values only, without considering medication use. The inverse associations were generally retained in these analyses. Specifically, after excluding medication users, inverse associations remained statistically significant when beef and mutton theoretically replaced refined grains, legumes, vegetables and fruits, poultry/fish/eggs, animal oil, sugar-sweetened beverages, and alcohol **(Supplementary Table 6)**. When MUO was redefined using measured blood pressure and fasting plasma glucose values only, inverse associations remained statistically significant for theoretical replacement of refined grains, legumes, vegetables and fruits, and sugar-sweetened beverages with beef and mutton **(Supplementary Table 7)**. **(****Line 221-227, Page 6; Line 285-291, Page 7)**

These additional analyses do not remove the limitations of the cross-sectional design, but they reduce the likelihood that the main finding is explained solely by diet changes after treatment or diagnosis. We have added the description of these analyses in the Statistical analysis section and reported the results in the Sensitivity analyses section and Supplementary Tables 6 and 7.

## Point 2

**Reviewer comment:** In the Results, the text states that after additional adjustment for total energy intake, inverse associations remained significant only when beef and mutton replaced refined grains, whole grains, and legumes. However, Supplementary Table 4 shows nine statistically significant substitutions. The text and Supplementary Table 4 do not agree.

**Response:** We thank the reviewer for identifying this inconsistency. We rechecked the Results section and Supplementary Table 4 and corrected the text so that it matches the supplementary table. The revised Results now state that, after additional adjustment for total energy intake, inverse associations remained statistically significant when beef and mutton theoretically replaced refined grains, whole grains, legumes, vegetables and fruits, poultry/fish/eggs, plant oil, animal oil, sugar-sweetened beverages, and alcohol (Supplementary Table 4). **(Line 280-283, Page 7)**

## Point 3

**Reviewer comment:** In Table 1, some continuous variables are presented differently for the two groups. For example, waist circumference and diastolic blood pressure are given as mean ± SD for the MHO group but as median (IQR) for the MUO group. The same variable should be summarized in the same way for both groups.

**Response:** We agree with the reviewer. We rechecked Table 1 and standardized the presentation of continuous variables so that each variable is summarized in the same format for both MHO and MUO groups. In the revised **Table 1**, all continuous variables are now consistently presented as median with the 25th and 75th percentiles [M (P25, P75)]. **(Line 555, Page 14)**

## Point 4

**Reviewer comment:** In Table 1, the marital status figures appear to be reversed: married is shown as about 5% and unmarried as about 92%, which is implausible for adults with a median age around 47-49 years. The reviewer asks whether the labels or values were switched.

**Response:** We thank the reviewer for carefully identifying this error. We rechecked the coding and labels for marital status and confirmed that the labels had been switched in **Table 1**. We have corrected the table. The revised Table 1 now shows that most participants were married: 404 (91.8%) in the MHO group and 613 (92.5%) in the MUO group, whereas 22 (5.0%) and 24 (3.6%) were unmarried, respectively. The corrected values are consistent with the age distribution of the study population. **(Line 555, Page 14)**

## Point 5

**Reviewer comment:** In Table 4, the cut-off values that define the quartiles of beef and mutton and dairy intake are not shown. Without these, readers cannot tell how much intake each quartile represents.

**Response:** We agree that the quartile definitions should be explicit. We have added the quartile cut-off values for beef and mutton and dairy intake to the note below **Table 4**. The revised note reports the cut-off values for the percentage of total energy intake contributed by beef and mutton and dairy, respectively. This addition allows readers to interpret the intake level represented by each quartile. **(Line 575-578, Page 16-17)**

**Response to Reviewer 2**

We thank the reviewer for the careful evaluation of our manuscript and for the constructive comments. We have revised the manuscript to clarify the study population, sample-size estimation, dietary assessment, interpretation of red-meat-related findings, and the limitations regarding generalizability and recall bias.

## Point 1

**Reviewer comment:** The manuscript is relevant to the broad audience (global population) since it discusses how beef and mutton consumption contributes to metabolically unhealthy or healthy obesity amongst other foods, such as whole grains, legumes among others. However, the sample population does not appear to represent the wider population; consequently, concluding that red meat consumption is not related to type 2 diabetes and cardiovascular diseases is not adequately supported.

**Response:** We thank the reviewer for this important comment. We agree that our sample should not be interpreted as representative of the global population. The present study was conducted among adults with obesity in Ordos, Inner Mongolia, a region with a distinctive livestock-oriented dietary structure, ethnic composition and food environment. Therefore, the findings are context-specific and should not be generalized to the wider global population or to adults without obesity. To avoid overstatement, we revised the Conclusion. **(Line 575-578, Page 16-17)** to clarify that our outcome was MUO among adults with obesity, not incident cardiometabolic disease, and that the observed associations should be interpreted as cross-sectional associations within specific food-group substitution contrasts rather than causal effects.

## Point 2

**Reviewer comment:** The author did not include the criteria used to select the sample, nor is the sample size calculation provided.

**Response:** We agree with the reviewer that the sampling criteria and sample-size estimation should be described more clearly. We added the sample-size calculation to the Study population section. These revisions clarify how the analytic sample was selected and show that the final sample size was sufficient for the planned cross-sectional analysis. **(Line 102-112, Page 3)**

## point 3

**Reviewer comment:** A further concern is that this study may have triggered biasness in data collection, since the participants were asked to recall their dietary patterns over a 30-day period. It is unlikely that they could accurately remember their exact meals, consumption frequency and portion sizes without retrieving from recorded records which were not mentioned in the paper.

**Response:** We thank the reviewer for raising this concern. We agree that self-reported dietary assessment is subject to recall bias and measurement error, particularly when participants are asked to report intake frequency and portion size retrospectively. We strengthened the Limitations section to explicitly acknowledge the possibility of recall bias. **(Line 369-373, Page 6)**

## point 4

**Reviewer comment:** The references needs to be revised to cover a period of 10 years or less.

**Response:** We thank the reviewer for this helpful suggestion. We reviewed and updated the reference list to ensure that recent evidence was adequately represented. In the revised manuscript, 36 of 43 references (83.7%) were published within the past 10 years, and 23 of 43 references (53.5%) were published within the past 5 years. **(Page 18)**

A small number of older references were retained because they are necessary for established diagnostic criteria, validated questionnaire processing, methodological background, or mechanistic context. For example, the NCEP ATP III report was retained because it provides the basis for the metabolic syndrome components used to define MUO, and the IPAQ-related reference was retained because it supports physical-activity data processing. Older mechanistic studies on vaccenic acid and conjugated linoleic acid were retained only as biological plausibility background, not as evidence for causal inference.
